# Impact of Tumor Budding in Head and Neck Cancers on Neck Lymph Node Metastasis and Prognosis

**DOI:** 10.3390/jcm14155224

**Published:** 2025-07-23

**Authors:** Oğuz Gül, Özlem Çelebi Erdivanlı, Mehmet Birinci, Suat Terzi, Metin Çeliker, Oğuzhan Okçu, Çiğdem Öztürk, Tuğba Yemiş, Fatma Beyazal Çeliker, Zerrin Özergin Coşkun, Engin Dursun

**Affiliations:** 1 Department of Otorhinolaryngology, Faculty of Medicine, Recep Tayyip Erdogan University, Rize 53200, Turkey; drogzgl@gmail.com (O.G.); mehmet.birinci@erdogan.edu.tr (M.B.); drsterzi@hotmail.com (S.T.); metin.celiker@erdogan.edu.tr (M.Ç.); tugba.yemis@erdogan.edu.tr (T.Y.); zerrin.coskun@erdogan.edu.tr (Z.Ö.C.); 2 Department of Pathology, Faculty of Medicine, Recep Tayyip Erdogan University, Rize 53200, Turkey; oguzhan.okcu@erdogan.edu.tr (O.O.); dr.ozturkcigdem@gmail.com (Ç.Ö.); 3 Department of Radiology, Faculty of Medicine, Recep Tayyip Erdogan University, Rize 53200, Turkey; fatma.bceliker@erdogan.edu.tr; 4 Department of Otorhinolaryngology, Faculty of Medicine, Lokman Hekim University, Ankara 06510, Turkey; engin.dursun@lokmanhekim.edu.tr

**Keywords:** tumor budding, head and neck cancer, head and neck squamous cell carcinoma, neck lymph node metastasis, prognosis, overall survival

## Abstract

**Background/Objectives:** Tumor budding (TB)—clusters of one to five tumor cells at the invasive front—has emerged as a prognostic marker in various cancers. Its prognostic value in head and neck squamous cell carcinoma (HNSCC) is unclear. **Methods:** We retrospectively analyzed 98 HNSCC patients. The tumor buds were counted on hematoxylin–eosin-stained sections as per the 2016 International Tumor Budding Consensus Conference (ITBCC) guidelines. An optimal cutoff was determined by ROC analysis using excisional lymph nodes and five-year overall survival (OS) as the endpoint, stratifying patients into low- (≤4 buds) and high-risk (>4 buds) groups. The associations with clinicopathological features, OS, and disease-free survival (DFS) were assessed using Kaplan–Meier curves and Cox regression. **Results:** Among the 98 patients (median follow-up 58 months, range 18–108), 32 (32.7%) died. The optimal TB cutoff was 4.5 (AUC 0.85, 95% CI 0.76–0.93). High TB was associated with poorer five-year OS (26.4% vs. 85.3%). Multivariate Cox regression identified TB and extranodal extension as independent predictors of OS (TB HR: 3.4, 95% CI 1.3–9.2, *p* = 0.013). In the laryngeal cancer subgroup, TB was associated with worse survival in the univariate analysis (HR 7.5, 95% CI 1.6–35.6, *p* = 0.011), though this was not significant in the multivariate modeling. High TB independently predicted neck lymph node metastasis (multivariate OR 4.9, 95% CI 1.2–20.5, *p* = 0.029), which was present in 65.8% of the high-TB vs. 31.7% of the low-TB patients. High TB correlated with advanced AJCC stage and lymphovascular invasion. No clinicopathological factors, including TB, independently predicted DFS, in either the full cohort or the laryngeal subgroup. **Conclusions:** High tumor budding denotes an aggressive HNSCC phenotype and may guide decisions on elective neck dissection. Its assessment is simple, cost-effective, and potentially valuable for routine pathology, pending external validation.

## 1. Introduction

Head and neck cancers represent a significant global burden. In 2018, these malignancies ranked as the sixth most common cancer worldwide, with approximately 890,000 new diagnoses and 450,000 deaths annually [1]. Recent projections estimate a 30% increase in head and neck cancer incidence by 2030 [2]. Squamous cell carcinoma (SCC) constitutes the most common histologic subtype of head and neck malignancies, representing over 90% of cases worldwide [2]. A precise prognostic assessment is crucial for effective management, as patient outcomes depend on multiple factors such as tumor stage, lymph node involvement, extranodal extension (ENE), negative surgical margins, lymphovascular invasion (LVI), perineural invasion (PNI), and distant metastasis, all of which also guide adjuvant therapy decisions [3].

Neck lymph node metastasis is a key determinant of both cancer stage and survival. Its presence often justifies elective neck dissection, even in cases without clinical evidence of nodal disease [4]. However, such surgical interventions carry risks of intraoperative and postoperative complications. Despite established prognostic factors, significant interpatient heterogeneity in treatment response and survival persists within similar clinical stages, underscoring the need for novel prognostic markers to improve survival predictions and personalize treatment strategies.

Tumor budding (TB) is defined as the presence of minute clusters of one to five tumor cells at the invasive margin [5,6]. Initially described in colorectal cancer, TB has emerged as a valuable prognostic marker due to its association with metastatic potential and aggressive tumor behavior [7]. High TB density is correlated with increased epithelial-to-mesenchymal transition, a critical process in tumor dissemination [8,9]. TB has been extensively studied in colorectal and breast cancers [10,11,12,13], and several recent investigations have evaluated its prognostic role in HNSCC—Pedersen et al. developed a semi-automated image-analysis model for occult nodal metastasis in oral tongue SCC [14], and Chatterjee et al. demonstrated TB’s correlation with nodal spread and poor outcomes in early oral cavity SCC [15]. However, no consensus exists regarding standardized TB risk classification or cutoff values in HNSCC, leaving an important gap that our study addresses.

While some researchers have proposed pancytokeratin immunohistochemistry for its sensitivity [16], recent evidence indicates that TB can be reliably assessed using standard hematoxylin and eosin (H&E) staining, obviating the need for advanced immunohistochemical techniques [17,18]. As H&E is routinely employed in all excised lymph node specimens, this makes TB scoring both practical and cost-effective for widespread clinical adoption. Building on the robust prognostic value established in colorectal and other cancers, we sought to evaluate whether TB—measured with routine methods—serves as a clinically relevant predictor of prognosis and neck lymph node metastasis in HNSCC.

This study investigates the prognostic value of TB for overall survival and its predictive value for neck lymph node metastasis in HNSCC, compared to established clinicopathological factors.

## 2. Materials and Methods

### 2.1. Study Design and Patient Selection

This retrospective study was conducted at Recep Tayyip Erdogan University Training and Research Hospital. The inclusion criteria were histologically confirmed head and neck squamous cell carcinoma, age 18 years or older, underwent surgical excision and neck dissection as the primary treatment at our institution, and a minimum follow-up period of 18 months. The exclusion criteria were non-squamous cell head and neck malignancy, recurrent head and neck cancer, incomplete medical records or inability to complete follow-up, refusal of treatment, and patients initially treated with non-surgical modalities (radiotherapy or chemoradiotherapy).

Between January 2013 and January 2022, 680 patients with HNSCC were initially assessed for eligibility. Of these, 179 had non-squamous cell carcinoma, and 17 cases had unknown primary head and neck cancer. Following the exclusion of 10 cases who rejected treatment, 38 cases who were treated in a different center, 8 cases where a pathology specimen was not found, 12 cases with incomplete survival records, and 318 patients who received radiotherapy, chemoradiotherapy, or total excision without neck dissection, 98 patients with histologically confirmed HNSCC who had undergone primary surgical excision and neck dissection remained in the final cohort.

The primary outcome was overall survival (OS), defined as the time from surgery to death from any cause. The secondary outcome was neck lymph node metastasis, defined as the presence of histopathologically confirmed metastatic lymph nodes in the neck dissection specimen. The exploratory outcomes included disease-free survival (DFS), defined as the time from surgery to recurrence or death, and subgroup analyses of laryngeal cancer cohort. For the exploratory subgroup analyses, the patients were stratified according to the primary anatomical site (larynx, oral cavity, skin, lips, oropharynx, and parotid) to assess the potential site-specific prognostic implications of TB.

### 2.2. Pathological Assessment

The pathological evaluation was performed by two experienced pathologists blinded to patient outcomes. They recorded the tumor size, the surgical margin status, LVI, PNI, the degree of differentiation, the depth of invasion, the presence of metastatic lymph nodes, ENE, anterior commissure involvement, thyroid cartilage involvement, extralaryngeal invasion, and tumor budding.

**Figure 1:**
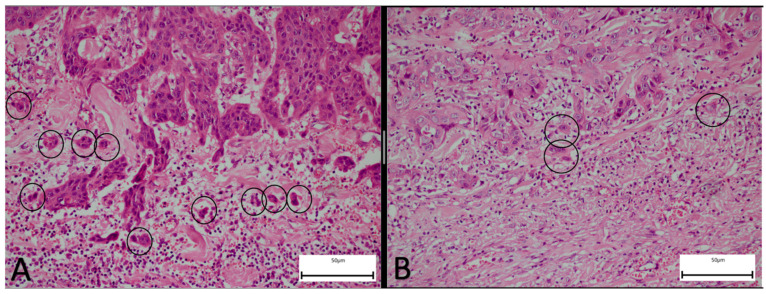
(**A**): High-magnification view of a head and neck squamous cell carcinoma with high tumor budding (H&E×200); (**B**): Head and neck squamous cell carcinoma with low tumor budding (H&E×200). Black circles indicate tumor buds, defined as isolated single tumor cells or small clusters at the invasive front.

For each case, at least three representative H&E-stained sections encompassing the tumor’s invasive front were examined. The tumor budding assessment was performed on the surgical specimen after the final pathological diagnosis was rendered but before the outcome data were analyzed; this ensured that the TB count was determined independently of the survival and nodal status. The tumor buds were counted according to the 2016 International Tumor Budding Consensus Conference (ITBCC) guidelines [7]. A tumor bud was defined as an isolated tumor cell or a cluster of one to four cells at the invasive front exhibiting both epithelial and mesenchymal features with no continuity with the main tumor mass. In cases where the independent counts differed by more than 10%, the slides were re-assessed jointly using a five-head Olympus BX53 microscope (Olympus Corporation, Center Valley, PA, USA) to achieve consensus. The staging was performed according to the 8th edition AJCC criteria. Representative histopathological images are provided in Figure 1.

### 2.3. Statistical Analysis

The data were analyzed using R version 4.4.0 (R Software Foundation, Vienna, Austria). The continuous variables were described as means ± standard deviations or medians with ranges, as appropriate; the categorical variables were summarized as frequencies and percentages. The distribution normality was assessed by the Kolmogorov–Smirnov test. For the prognostic stratification of TB, a receiver operating characteristic curve was generated using five-year OS as the endpoint; the cutoff maximizing the Youden index was used to dichotomize the TB into low-risk and high-risk groups.

The factors associated with OS were evaluated by univariate and multivariate Cox proportional hazards regression; hazard ratios and 95% confidence intervals were reported. The survival distributions were estimated using the Kaplan–Meier method and compared by log-rank test. The associations between TB and other clinicopathological variables with neck lymph node metastasis were assessed using univariate and multivariate logistic regression models; odds ratios and 95% confidence intervals were reported.

The exploratory analyses included Cox proportional hazards regression, Kaplan–Meier estimation, and log-rank test for DFS. Additionally, subgroup analyses were applied within the laryngeal cancer cohort to assess the prognostic significance of TB and other clinicopathological risk factors for OS and DFS. All statistical tests were two-sided, and a *p*-value < 0.05 was considered statistically significant.

## 3. Results

### 3.1. Patient Cohort and Survival Outcomes

Initially, 680 patients diagnosed with head and neck malignancies were identified; after applying stringent inclusion and exclusion criteria, a final cohort of 98 patients with histologically confirmed HNSCC was assembled, with a median follow-up duration of 58 months (range: 18–108 months). Of these, 48 (49%) had laryngeal primaries, 28 (28.6%) had oral cavity primaries, and the remainder (*n* = 22, 22.4%) were distributed among skin, lips, oropharynx, and parotid. Among the 98 patients, 32 (32.7%) died during the median follow-up of 58 months (range: 18–108).

### 3.2. Tumor Budding Grouping

**Figure 2:**
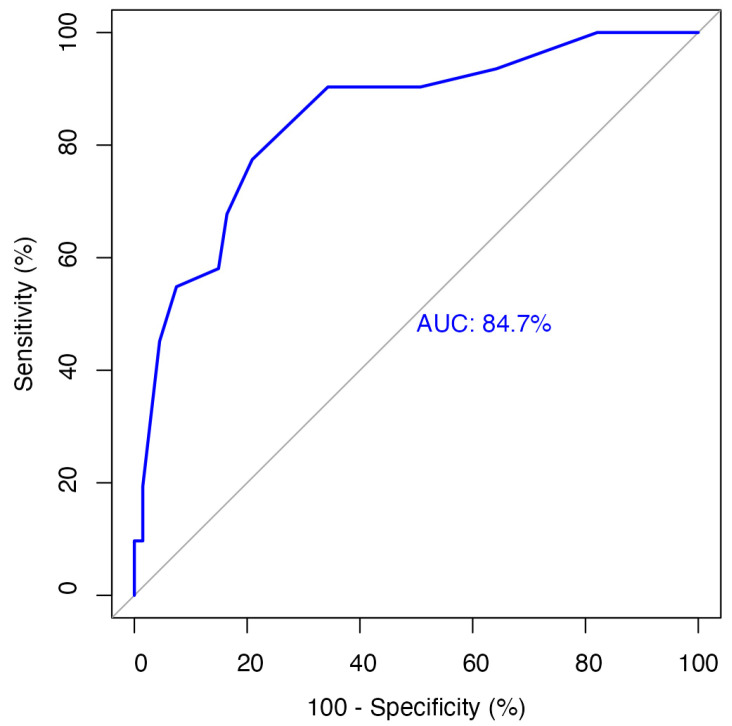
Receiver operating characteristic curve demonstrating the diagnostic performance of tumor budding in predicting overall survival in HNSCC patients. The analysis yielded an area under the curve of 0.847 (95% CI: 0.763–0.931) with an optimal cutoff value of 4.5, achieving a sensitivity of 77.4% and a specificity of 79.1% (*p* < 0.001).

The optimal cutoff value for tumor budding in the excisional specimens was 4.5, determined by receiver operating characteristic analysis for five-year overall survival (sensitivity 77%, specificity 79%) (Figure 2). Accordingly, the patients were categorized as low TB (≤4 buds) or high TB (>4 buds).

### 3.3. Patient and Tumor Characteristics

Table 1 summarizes the demographic and histopathological characteristics of the low-risk and high-risk groups. Notably, a statistically significant association was observed between high tumor budding and adverse prognostic markers such as lymphovascular invasion, the presence of metastatic lymph nodes, and perinodal spread (*p* < 0.05). Additionally, Table 2 demonstrates that the proportion of patients with high tumor budding increased significantly with advancing AJCC 8th edition stage (*p* < 0.05).

### 3.4. Primary Outcome: Overall Survival Analysis

**Figure 3:**
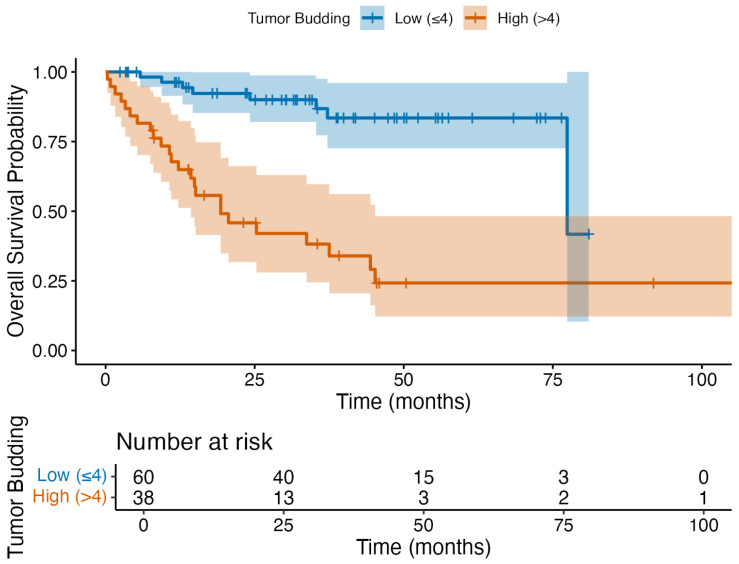
Kaplan–Meier survival curves comparing overall survival between patients with low tumor budding (≤4) and high tumor budding (>4). The high-risk group demonstrated significantly reduced overall survival (log-rank *p* < 0.05). Please note that the abrupt drop in the low tumor budding group after 75 months reflects a late event, with only three low-TB and two high-TB patients remaining at risk, as shown in the risk table.

Cox regression demonstrated that both tumor budding and extranodal extension were independent predictors of overall survival (Table 3). The five-year OS was 85.3% in patients with low TB (*n* = 52/60), while it was 26.4% (*n* = 10/38) in those with high TB, as plotted in the Kaplan–Meier survival curve (Figure 3). These findings support the prognostic value of TB for OS in HNSCC.

### 3.5. Secondary Outcome: Neck Lymph Node Metastasis

Neck metastasis was present in 65.8% (*n* = 25/38) of the patients in the high-TB group and 31.7% (*n* = 19/60) in the low-TB group. Logistic regression revealed that high TB was an independent predictor of neck lymph node metastasis, with an odds ratio of 4.9 (*p* = 0.029, Table 4). The median overall survival was 35 months in the low-TB group compared to 23 months in the high-TB group, reflecting the substantial prognostic impact of tumor budding.

### 3.6. Exploratory Outcomes

**Figure 4:**
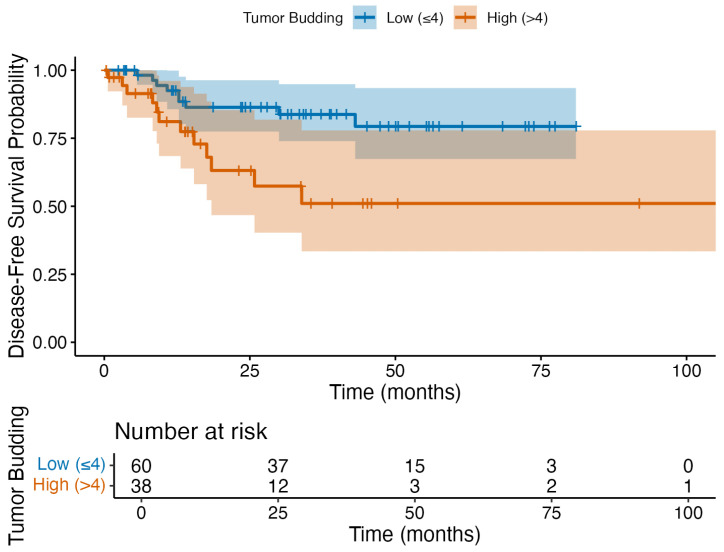
Kaplan–Meier curves illustrating the disease-free survival (DFS) in HNSCC patients grouped by tumor budding status (low: ≤4; high: >4). No statistically significant difference in the DFS was observed between the groups (log-rank *p* > 0.05). Please note that the number at risk fell to three or fewer after 75 months, so estimates beyond this timepoint should be interpreted with caution.

In the univariate Cox regression, metastatic lymph node involvement and extranodal extension were significantly associated with a shorter DFS (Table 5). However, in the multivariate analysis, none of the evaluated risk factors, including tumor budding, perineural invasion, lymphovascular invasion, metastatic lymph node involvement, and extranodal extension, remained independently associated with the DFS (*p* > 0.05). The Kaplan–Meier survival curve further illustrates that the DFS did not differ significantly between the low (≤4) and high (>4) tumor budding groups (log-rank *p* > 0.05; Figure 4).

In the laryngeal cancer subgroup (*n* = 48), the univariate Cox regression identified high tumor budding (HR 7.5, 95% CI 1.6–35.6, *p* = 0.011), thyroid internal cartilage involvement, metastatic lymph node involvement, and extranodal extension as significant risk factors for reduced disease-free survival (Table 6). However, in the multivariate analysis, none of these variables retained statistical significance as independent predictors of DFS (all *p*-values > 0.05).

**Figure 5:**
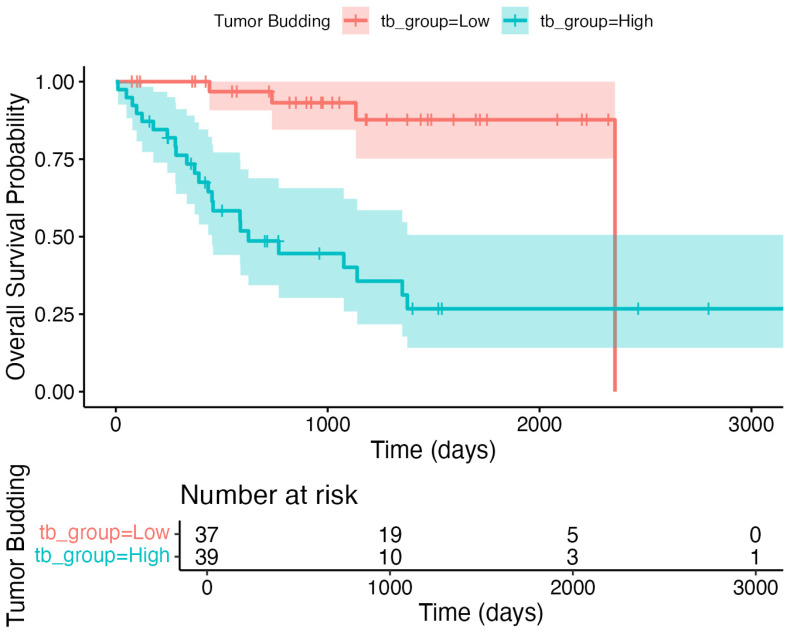
Overall survival in the larynx  +  oral cavity sensitivity cohort (*n* = 76), stratified by tumor budding status. Kaplan–Meier curve for overall survival by tumor budding group using the Youden-derived cut-point of 3.5 buds (low: ≤3 buds, *n* = 37; high: ≥4 buds, *n* = 39). High tumor budding was associated with significantly poorer OS (log-rank *p* < 0.001). Tick marks indicate censored observations. Number at risk is shown below the *x*-axis; after ~2000 days (~66 months), fewer than 10 patients remained at risk, so estimates beyond this time should be interpreted with caution.

**Figure 6:**
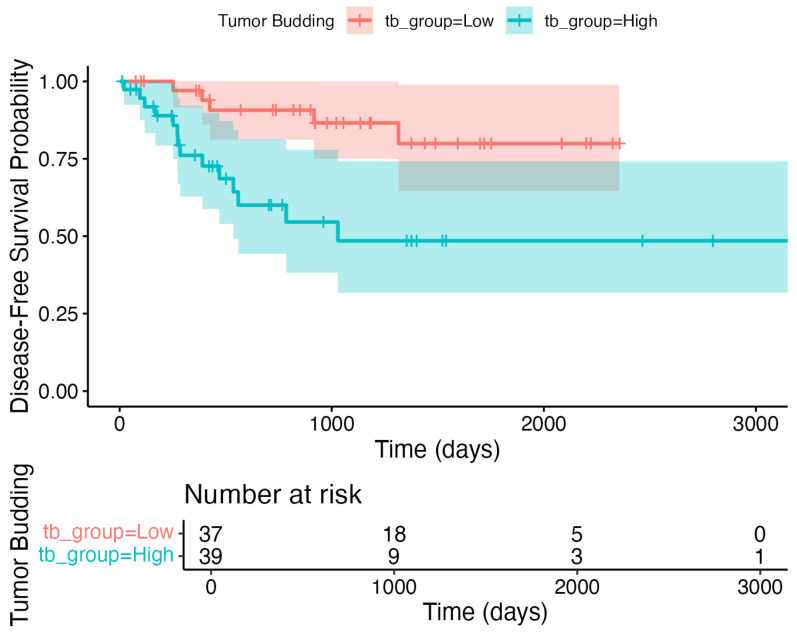
Disease-free survival in the larynx  +  oral cavity sensitivity cohort (*n* = 76), stratified by tumor budding status. Kaplan–Meier curve for disease-free survival by tumor budding group using the same 3.5-bud threshold (low: ≤3 buds, *n* = 37; high: ≥4 buds, *n* = 39). High tumor budding correlated with significantly reduced DFS (log-rank *p* < 0.001). Tick marks denote censored observations. Number at risk is shown below; because fewer than 10 patients were at risk after ~2000 days, DFS estimates beyond that time should be interpreted with caution.

To address the potential anatomic heterogeneity, we repeated our core analyses in the restricted cohort of laryngeal and oral cavity SCC (*n* = 76). Receiver operating characteristic analysis of tumor budding for five-year overall survival yielded an AUC of 0.837 (95% CI 0.740–0.934; *p* < 0.001). Youden’s index in this subgroup identified 3.5 buds as the optimal threshold, which, when applied, divided the cohort into low TB (*n* = 37) and high TB (*n* = 39). In the multivariable Cox regression, high TB remained a strong independent predictor of poorer OS (HR 6.26; 95% CI 2.13–18.37; *p* = 0.0008), closely mirroring the effect size in the full cohort. The multivariable logistic regression similarly confirmed that high TB significantly predicted neck lymph node metastasis (OR 7.16; 95% CI 1.39–36.78; *p* = 0.018). The Kaplan–Meier analyses again demonstrated markedly reduced OS and DFS in the high-TB group (log-rank *p* < 0.001 for both; Figure 5 and Figure 6, respectively).

## 4. Discussion

Recognizing the absence of a definitive consensus, our study aimed to establish a cutoff value for TB risk classification in head and neck cancer patients. The cutoff value for tumor budding was determined from excisional specimens, as this approach is consistent with prior studies and ensures a comprehensive assessment of the invasive tumor front. While incisional (preoperative) TB was also evaluated, its reliability is limited by sample size and the potential underrepresentation of tumor heterogeneity. Therefore, excisional TB currently provides the most accurate prognostic information, although future studies should clarify the role and optimal cutoff of incisional TB in preoperative settings.

We employed ROC curve analysis, leveraging overall survival data as the primary outcome measure. This analysis identified a cutoff point of 4.5 tumor buds, stratifying patients into low-risk (≤4 buds) and high-risk (>4 buds) categories. Notably, this cutoff value aligns with the established thresholds reported in the literature [5,11], lending further credence to its validity. Given that neck dissection carries significant morbidity, the ability to predict occult neck metastasis through the preoperative biopsy evaluation of TB may allow clinicians to tailor surgical management more effectively. Our results suggest that the quantification of TB, alongside other prognostic factors, could eventually guide decisions on elective neck dissection.

Recognizing the ongoing debate surrounding tumor budding quantification in the literature, we opted to employ the standardized counting protocol outlined in the 2016 International Tumor Budding Consensus Meeting [7]. This established methodology promotes consistency and facilitates reliable comparison with other studies adopting the same criteria.

In head and neck cancers, metastasis to the cervical lymph nodes is one of the most crucial parameters determining the prognosis of the patient [19,20]. Particularly in cases without clinical lymph node involvement (cN0), various approaches are taken in neck treatment. The literature suggests that elective neck dissection improves both the overall survival and disease-free survival [21,22,23,24]. All these studies underscore the paramount significance of determining the course of neck treatment at the outset of the initial diagnosis. On the other hand, 70–80% of patients undergoing elective neck dissection do not exhibit pathological lymph node involvement in the neck [25]. This operation, however, can lead to morbidities, especially in the shoulder and neck regions [26]. Identifying hidden lymph node metastases in the neck in head and neck cancers not only diminishes morbidity by avoiding unnecessary neck dissections but also, when essential, improves overall survival. Hence, there is ongoing investigation into histopathological prognostic criteria aimed at identifying clinically occult lymph node metastasis in the neck for patients with head and neck cancers. In this context, we believe that the results of our study could be beneficial.

There is a body of literature suggesting that tumor budding may be an indicator of metastasis to the neck lymph nodes [14,15]. In a study that investigated the prognostic criteria (TB, LVI, PNI, depth of tumor invasion, tumor stromal response, worst pattern of invasion) associated with neck lymph node metastasis in oral cavity cancers, TB and worst pattern of invasion were found to be significantly associated with neck nodal metastasis in the multivariate analysis [27]. In our study, when we examined the relationship between neck lymph node metastasis and tumor budding, we found that there was a significant difference (*p* < 0.05) between the high-risk group and the low-risk group. In total, 65.8% of the patients with a high TB count and 31.7% of the patients with a low TB count had metastatic lymph nodes in the neck. We also examined the risk factors that may increase neck lymph node metastasis and found that TB, LVI, PNI, and a tumor invasion depth of more than 10 mm were significant. However, when these risk factors were evaluated via a multivariate model in logistic regression analysis, only TB and LVI were found to be independent risk factors. Our results showed that tumor budding alone increased the risk of lymph node metastasis to the neck by four-fold. Importantly, when we restricted our analyses to the 76 patients with laryngeal and oral cavity SCC, tumor budding maintained its strong prognostic and predictive value (five-year OS AUC = 0.837; HR high vs. low = 6.26, *p* = 0.0008; OR for nodal metastasis = 7.16, *p* = 0.018). This sensitivity analysis confirms that our bud-count cut-point and its association with survival and neck metastasis are not driven by non-aero-digestive subsites but instead reflect robust biological behavior within the primary aero-digestive tract. Based on these results, information about TB may help clinicians in treatment planning. Therefore, it may be clinically important for patients in whom clinicians hesitate to perform neck dissection.

The prognostic significance of extranodal extension (ENE) in head and neck cancers led to the revision of nodal staging in the 8th edition of AJCC [28]. The assessment of ENE relies on pathological samples, while preoperative detection efforts have predominantly focused on radiological imaging [29]. However, due to the inconsistent reliability of radiological data, studies have been conducted to identify ENE using histopathological markers [30]. This study contributes to the growing evidence by demonstrating a significant correlation between the high-TB group and ENE. Furthermore, our study employed routine H&E staining to assess TB, offering a practical approach that may complement the more specialized or ancillary techniques reported in other investigations.

Our analysis revealed no statistically significant association between TB and several clinicopathological factors, including gender, degree of tumor differentiation, tumor subsite within the head and neck region, adjuvant treatment received after surgery, and metastatic status. This lack of association, particularly with regard to adjuvant therapy, mitigates the potential bias arising from the well-documented positive impact of such treatments on survival, which could otherwise confound the evaluation of survival differences between TB subgroups. While the sample size within individual head and neck cancer subsites may be considered modest, the absence of significant associations across diverse subgroups suggests that the combined analysis of all head and neck cancers in our study may be a valid approach.

Our study builds upon the limited existing literature investigating the prognostic significance of TB in laryngeal cancer. While Sarioglu et al. demonstrated a correlation between high TB and distant metastasis in laryngeal cancer, their study lacked data on survival outcomes [31]. Similarly, Ekmekçi et al. observed associations between TB and adverse clinicopathological factors like PNI, LVI, and neck node metastasis, but the survival data were not reported [32]. Karpathiou et al. found a statistically significant impact of TB on survival in head and neck cancers, including laryngeal and pharyngeal subtypes, but their heterogeneous patient population and varied methodologies limited the generalizability of their findings [33]. Our study not only strengthens the evidence for TB as a prognostic criterion in laryngeal cancers, impacting both overall and disease-free survival, but also represents the first investigation to demonstrate TB as the single most significant predictor of overall survival in multivariate analysis.

The study has several limitations, including its retrospective study, the single-center design, and the limited number of patients and subgroups within the head and neck cancer category. Besides, certain parameters, such as the worst invasion pattern and tumor stromal response, were not investigated concerning the development of metastatic lymph nodes, aside from tumor budding.

Tumor budding serves as a valuable marker of biological aggressiveness in head and neck cancers, promoting invasion and metastasis. Its ease of identification stems from the simple counting of buds in readily available H&E–stained sections. This simplicity, coupled with the widespread availability and low cost of H&E staining, makes TB a particularly attractive candidate for new prognostic markers in clinical practice. While the existing literature highlights the potential of TB in prognostic assessment, methodological inconsistencies present challenges to its widespread adoption. Despite these challenges, a growing body of research demonstrates a significant association between high TB scores and adverse outcomes, including disease-free survival, overall survival, disease-specific survival, and lymph node metastasis. This underscores the promise of TB as a prognostic tool, and further efforts should be directed towards standardizing the methodology, validating the optimal cutoff values, and investigating the integration of TB into clinical decision-making.

## 5. Conclusions

In this single-center study spanning nearly a decade, we identified TB as a robust histopathological marker of aggressive disease biology in HNSCC. The depth and integrity of our dataset—comprising standardized TB scoring, complete surgical treatment, and long-term follow-up of up to 108 months—provide a compelling foundation for the observed associations and firmly support the prognostic significance of TB, particularly in cN0 patients and those with laryngeal SCC.

Our findings lay the groundwork for future prospective studies evaluating TB on incisional biopsy specimens. If validated, this approach could enable the early prediction of neck metastasis and inform more aggressive or targeted treatment strategies, ultimately reducing overtreatment and the related healthcare costs.

## Figures and Tables

**Table 1:** Demographic and histopathological characteristics of 98 HNSCC patients stratified by tumor budding as low (≤4) vs. high (>4). Values are presented as median (min–max) or *n* (%).

Variables	Total (*n* = 98)	Tumor Budding Count		*p*-Value
		Low (≤4) (*n* = 60)	High (>4) (*n* = 38)	
Age	65 (19–92)	63 (19–92)	68 (33–92)	0.105
Gender				0.327
Female	19 (19.4%)	14 (23.3%)	5 (13.2%)	
Male	79 (80.6%)	46 (76.7%)	33 (86.8%)	
Primary Region				0.323
Larynx	48 (49%)	29 (48.3%)	19 (50%)	
Oral cavity	28 (28.6%)	19 (31.7%)	9 (23.7%)	
Skin	10 (10.2%)	3 (5%)	7 (18.5%)	
Lips	7 (7.1%)	6 (10%)	1 (2.6%)	
Oropharynx	3 (3.1%)	2 (3.3%)	1 (2.6%)	
Parotid	2 (2%)	1 (1.7%)	1 (2.6%)	
Tumor grade				0.189
Mild	8 (8.2%)	7 (11.7%)	1 (2.6%)	
Moderate	85 (86.7%)	51 (85%)	34 (89.5%)	
Severe	5 (5.1%)	2 (3.3%)	3 (7.9%)	
Perineural invasion	42 (42.9%)	21 (35%)	21 (55.3%)	0.077
Lymphovascular invasion	37 (37.8%)	17 (28.3%)	20 (52.6%)	0.028
Depth of Invasion	9 (2–25)	9 (2–22)	11 (2–25)	0.186
≤5 mm	20 (23.8%)	14 (26.4%)	6 (19.4%)	0.104
5–10 mm	25 (29.8%)	19 (35.8%)	6 (19.4%)	
>10 mm	39 (46.4%)	20 (37.7%)	19 (61.3%)	
Anterior commissure involvement	25 (89.3%)	14 (82.4%)	11 (100%)	0.258
Inner perichondrium involvement *	30 (62.5%)	15 (51.7%)	15 (78.9%)	0.110
External perichondrium involvement *	19 (39.6%)	9 (31.0%)	10 (52.6%)	0.232
Extralaryngeal spread	12 (25.5%)	5 (17.2%)	7 (38.9%)	0.168
Metastatic lymph node	44 (44.9%)	19 (31.7%)	25 (65.8%)	0.002
Extranodal extension	24 (24.5%)	5 (8.3%)	19 (50%)	<0.001
Post-op adjuvant chemotherapy	34 (35.8%)	17 (29.3%)	17 (45.9%)	0.153
Post-op adjuvant radiotherapy	50 (52.6%)	26 (44.8%)	24 (64.9%)	0.090
Metastasis	4 (4.1%)	1 (1.7%)	3 (7.9%)	0.296

* Thyroid cartilage involvement.

**Table 2:** Distribution of tumor (T), nodal (N), and overall AJCC 8th edition stages in HNSCC patients according to tumor budding status. Values are presented as *n* (%) or median (min–max).

Variables	Total (*n* = 98)	Tumor Budding Count		*p*-Value
		Low (≤4) (*n* = 60)	High (>4) (*n* = 38)	
T Category				0.021
T1	16 (16.3%)	15 (25%)	1 (2.6%)	
T2	28 (28.6%)	17 (28.3%)	11 (28.9%)	
T3	34 (34.7%)	19 (31.7%)	15 (39.5%)	
T4	20 (20.4%)	9 (15%)	11 (28.9%)	
N Category				<0.001
N0	54 (55.1%)	41 (68.3%)	13 (34.2%)	
N1	13 (13.3%)	8 (13.3%)	5 (13.2%)	
N2	9 (9.2%)	7 (11.7%)	2 (5.3%)	
N3	22 (22.4%)	4 (6.7%)	18 (47.4%)	
N2	*n* = 9	*n* = 7	*n* = 2	0.413
N2A	2 (22.2%)	1 (14.3%)	1 (50%)	
N2B	4 (44.4%)	3 (42.9%)	1 (50%)	
N2C	3 (33.3%)	3 (42.9%)	-	
AJCC 8 Stage				0.006
Stage 1	12 (12.2%)	12 (20%)	-	
Stage 2	17 (17.3%)	11 (18.3%)	6 (15.8%)	
Stage 3	27 (27.6%)	18 (30%)	9 (23.7%)	
Stage 4	42 (42.9%)	19 (31.7%)	23 (60.5%)	

**Table 3:** Univariate and multivariate Cox regression of risk factors for overall survival in HNSCC.

	Univariate		Multivariate	
Variables	Hazard Ratio (95% CI)	*p*-Value	Hazard Ratio (95% CI)	*p*-Value
Tumor budding (low–high)	6.9 (3.1–15.4)	<0.001	3.4 (1.3–9.2)	0.013
Perineural invasion	3.2 (1.6–6.7)	0.002	1.3 (0.5–3.6)	0.626
Lymphovascular invasion	2.4 (1.2–4.7)	0.017	0.3 (0.1–1.0)	0.051
Metastatic lymph node involvement	3.5 (1.6–7.6)	0.001	1.1 (0.2–5.1)	0.901

**Table 4:** Univariate and multivariate logistic regression of risk factors associated with neck lymph node metastasis in HNSCC.

	Univariate		Multivariate	
Variables	Odds Ratio (95% CI)	*p*-Value	Odds Ratio (95% CI)	*p*-Value
Tumor budding (low–high)	4.2 (1.8–9.8)	0.001	4.9 (1.2–20.5)	0.029
Perineural invasion	4.1 (1.8–9.7)	0.001	2.2(0.5–10.0)	0.304
Lymphovascular invasion	57.8 (14.8–225.5)	<0.001	74.2 (12.9–426.8)	<0.001
Depth of invasion > 10 mm	5.4 (1.6–17.9)	0.006	0.5 (0.1–2.5)	0.303

**Table 5:** Univariate and multivariate Cox regression of risk factors for disease-free survival in HNSCC.

	Univariate		Multivariate	
Variables	Hazard Ratio (95% CI)	*p*-Value	Hazard Ratio (95% CI)	*p*-Value
Tumor budding (low–high)	2.2 (0.9–5.3)	0.071		
Perineural invasion	1.3 (0.5–3.0)	0.600		
Lymphovascular invasion	1.4 (0.6–3.4)	0.416		
Metastatic lymph node involvement	3.1 (1.2–8.1)	0.018	1.8 (0.5–6.3)	0.379
Extranodal extension	3.8 (1.6–9.0)	0.002	2.6 (0.8–8.2)	0.100

**Table 6:** Univariate and multivariate Cox regression analysis of clinicopathological risk factors affecting overall survival in laryngeal cancer patients (*n* = 48).

	Univariate		Multivariate	
Variables	Hazard Ratio (95% CI)	*p*-Value	Hazard Ratio (95% CI)	*p*-Value
Tumor budding (low–high)	7.5 (1.6–35.6)	0.011	3.5 (0.6–19.0)	0.142
Thyroid internal cartilage involvement	6.5 (1.8–51.3)	0.046	3.8 (0.5–31.0)	0.214
Metastatic lymph node involvement	5.8 (1.2–27.4)	0.026	1.7 (0.2–12.9)	0.596
Extranodal extension	6.2 (1.7–22.0)	0.005	2.4 (0.5–12.1)	0.294

## Data Availability

The raw data supporting the reported results and the R source files used during the analysis will be made available by the authors on reasonable request.

## Abbreviations

The following abbreviations are used in this manuscript:

TB	Tumor budding
HNSCC	Head and neck squamous cell carcinoma
ITBCC	International Tumor Budding Consensus Conference
ROC	Receiver operating characteristics
OS	Overall survival
DFS	Disease-free survival
AUC	Area under the curve
CI	Confidence interval
HR	Hazard ratio
OR	Odds ratio
AJCC	American Joint Committee on Cancer
ENE	Extranodal extension
LVI	Lymphovascular invasion
PNI	Perineural invasion
